# Air pollution from livestock farms and lung function decline in neighboring residents over 7 years

**DOI:** 10.1097/EE9.0000000000000479

**Published:** 2026-04-15

**Authors:** Warner van Kersen, Myrna M. T. de Rooij, Bernadette Aalders, Kaitlin Prins, Floor Borlée, Joris C. Yzermans, Joke W. B. van der Giessen, Dick Heederik, Mary B. Rice, Lidwien A. M. Smit

**Affiliations:** a Institute for Risk Assessment Sciences (IRAS), Utrecht University, Utrecht, the Netherlands; b Netherlands Expertise Centre for Occupational Respiratory Disorders, Utrecht, the Netherlands; c Netherlands Institute for Health Services Research, Utrecht, the Netherlands; d Centre for Infectious Disease Control, National Institute for Public Health and the Environment, Bilthoven, the Netherlands; e Division of Pulmonary, Critical Care and Sleep Medicine, Beth Israel Deaconess Medical Center, Boston, Massachusetts

**Keywords:** Livestock farming, Air pollution, Lung function

## Abstract

**Background:**

Longitudinal studies investigating air pollution from livestock farms and respiratory health effects in neighboring residents are lacking. The aim of this study was to assess the relationship between residential livestock farm exposures and lung function decline over a 7-year period in people living in livestock-dense rural areas in the Netherlands.

**Methods:**

Spirometry was performed in 2014/2015 and 2021/2022 for 847 adults (28–80 years of age at follow-up). We analyzed the annual rate of change in forced expiratory volume in 1 second (FEV_1_), forced vital capacity (FVC), tiffeneau index (FEV_1_/FVC) , peak expiratory flow, and maximal mid-expiratory flow in relation to long-term exposure to livestock farming-emitted endotoxin and particulate matter <10 µm (PM_10_) at the home address, which was predicted by dispersion modeling at baseline. Data analysis was performed using generalized additive models with nonlinear terms for exposure, adjusting for potential confounders.

**Results:**

No associations were identified between residential exposure to livestock-related endotoxin or PM_10_ and the annual rate of change in lung function (*P* > 0.05). Adjusted models showed that participants with a farm childhood had larger annual decreases in FEV_1_ (−5.63 ml/year, *P* = 0.018) and maximal mid-expiratory flow (−11.15 ml/second per year, *P* = 0.032), compared with those without a farm childhood. However, average baseline spirometry was higher in participants with a farm childhood compared with those without.

**Conclusion:**

Our longitudinal study did not find evidence for a relationship between air pollution from livestock farms and lung function decline in neighboring residents. Longitudinal studies with a greater number of observations across the life course are needed to gain deeper insights into lung function trajectories and to assess the impact of livestock-related air pollution in rural populations.

**What this study adds**

This longitudinal study assesses the impact of livestock-related air pollution on lung function decline in residents of a rural, livestock-dense area in the Netherlands. Over a 7-year period, no association could be identified between exposure to livestock-emitted endotoxin and PM_10_ and lung function decline. However, individuals with a farm childhood showed a slightly greater decline in some lung function measures, despite having better baseline lung function. These findings highlight the need for life course studies to better understand respiratory health trajectories in rural populations exposed to agricultural air pollution.

## Introduction

Ambient air pollution has been associated with adverse effects on lung function throughout the course of human life.^1^ In adults, longitudinal studies have found that concentrations of ambient pollutants as well as proxies of exposure (e.g., distance to the nearest road) are associated with acceleration of lung function decline – a process that naturally occurs with age.^2–7^ Studies focusing on air pollution from livestock farms indicated lung function deficits in nearby residents,^8–16^ but most evidence comes from cross-sectional research or stems from panel studies with short follow-up times. As a result, the long-term effects of exposure to air pollutants from livestock farms on lung function decline remain poorly understood.

Livestock farms emit complex mixtures of particulate matter (PM) and gaseous pollutants such as ammonia. This primary PM emitted by livestock farming is distinctly different from PM emitted by other major sources like industry and traffic; it has a predominantly organic composition and contains high levels of microorganisms and microbial components such as endotoxin, a Gram-negative bacterial cell wall component with high toxic potential.^17^ Ammonia (NH_3_) is an irritant gas that is formed from manure and emitted by livestock farms into the atmosphere. As an important precursor for secondary inorganic aerosols, it contributes greatly to ambient PM with an aerodynamic diameter of less than 2.5 µm (PM_2.5_), which can be transported over long distances.^18^

The aim of this study is to investigate the relationship between air pollution from livestock farms and long-term changes in lung function in a general, nonfarming, rural population in the Netherlands. This relatively elderly cohort was examined by spirometry for the first time in 2014–2015 (baseline),^8^ and followed up in 2021–2022 (participant’s mean age 63.3 years). The study performed at baseline found short-term exposure, expressed as week-average NH_3_ concentrations in the week before examination, to be negatively associated with forced expiratory volume in 1 second (FEV_1_) and maximal mid-expiratory flow (MMEF). Additionally, a higher number of livestock farms within 1km of the home address was associated with a lower MMEF. We hypothesize that air pollution from livestock farms is associated with accelerated lung function decline in our population. To test this hypothesis, the rate of change in lung function over the follow-up period was analyzed in relation to long-term residential exposure to livestock farming emitted particulate matter <10 µm (PM_10_) and endotoxin.

## Materials and methods

### Study population and design

**Figure 1.**
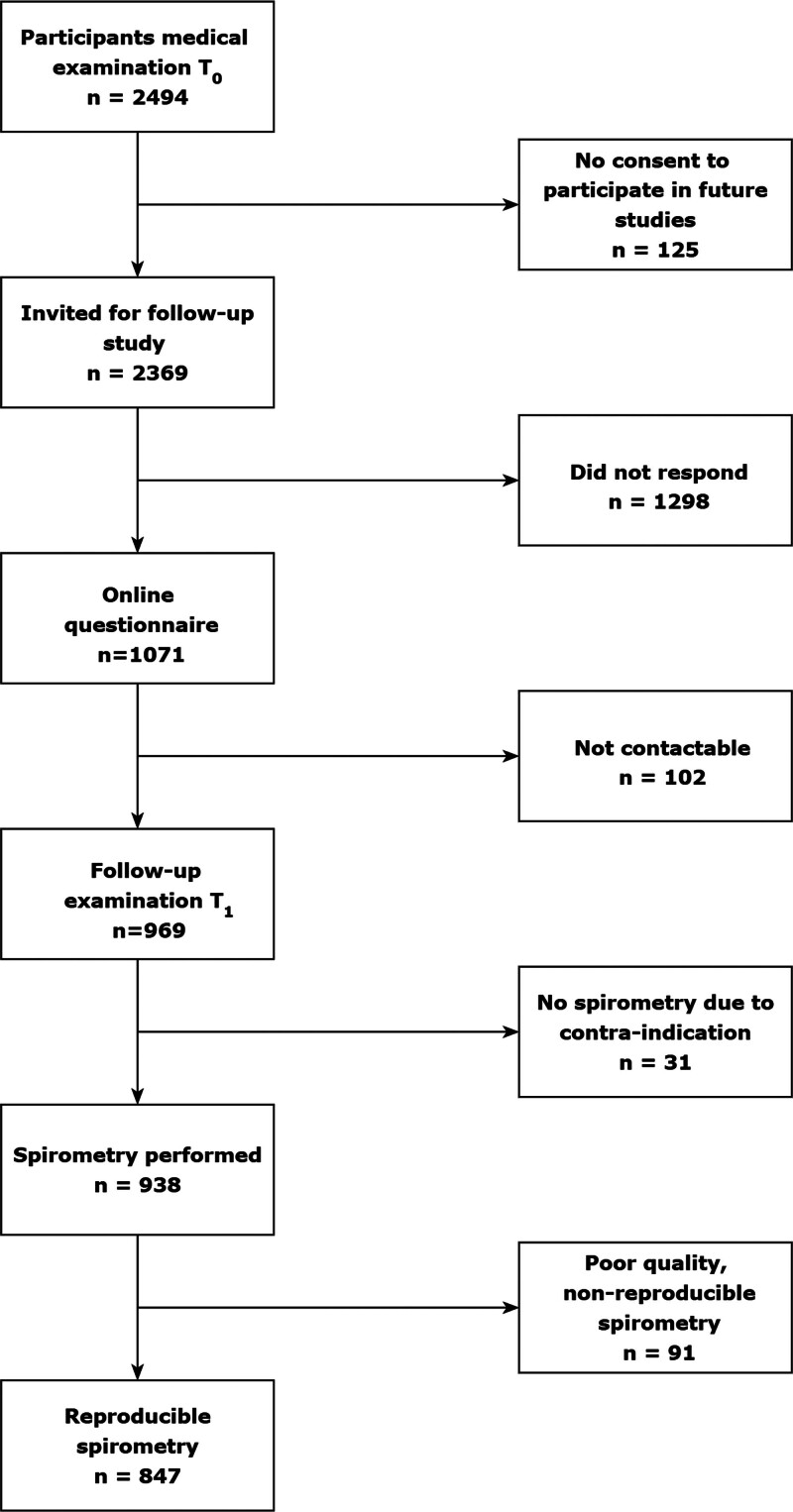
Flowchart of the study population.

This present prospective cohort study describes a 7-year follow-up between two medical examinations of participants from the Livestock Farming and Neighboring Residents’ Health research program (Dutch: Veehouderij en Gezondheid Omwonenden – VGO).^8^ The original design and recruitment of the VGO cohort have been described in detail before.^11,19^ Briefly, in 2012, 14,882 individuals completed a survey among patients (aged 18–70 years) of 21 general practitioners in a livestock-dense area in the southeast of the Netherlands. Subsequently, a medical examination was conducted between March 2014 and February 2015 in 2,494 subjects that agreed to participate and were not working or living on a farm. Of this study population, 2,369 participants had signed informed consent to be contacted for future research. These participants were invited for follow-up by mail, with maximally two reminders, between September 2020 and May 2022. In total, 969 participants (response rate 41%) underwent a follow-up examination during a home visit between August 2021 and July 2022. A flow chart of the study population can be found in Figure 1. According to health safety protocols, home visits were performed by trained fieldworkers who were COVID-19 negative (daily self-testing). Participants were phoned by the fieldworker to inquire about their COVID-19 status before the visit, which was rescheduled in case of confirmed or suspected COVID-19. All participants gave written informed consent, and the study protocols of the initial (13/533) and present study (19–536/D) were approved by the Medical Ethical Committee of the University Medical Centre Utrecht.

### Respiratory health assessment

Both at baseline (T_0_ = 2014–2015) and at 7-year follow-up (T_1_ = 2021–2022), population characteristics were collected by questionnaires. Smoking status was categorized into never-, current-, and former smokers. Atopy was defined, at baseline, as the presence of specific serum immunoglobulin E antibodies to one or more common allergens (house dust mite, grass, cat, and dog) and/or a total immunoglobulin E higher than 100 IU/ml. At both examinations, in nonclinical settings, prebronchodilator FEV_1_ (l), forced vital capacity (FVC, l), FEV_1_/FVC, MMEF (l/s), and Peak Expiratory Flow (PEF, l/s) were measured by spirometry and expressed as both continuous variables and percent-predicted values using the Global Lung Function Initiative (GLI) reference equations.^20^ Only reproducible spirometry measurements that conform to European Respiratory Society and American Thoracic Society standards were included in the analysis.^21^ We calculated the annual rate of change in lung function parameters between examinations as the difference between T_1_ and T_0_ spirometry values, divided by the time period between T_0_ and T_1_ dates (e.g., 7.2 years).^6^ Definitions of other characteristics can be found in Table 1. Details on secondary outcomes can be found in the Supplementary methods; https://links.lww.com/EE/A421.

**Table 1.** VGO study participant characteristics at 7-year follow-up

	Overall (N = 847)
Age (year)	63.3 (10.0)
Female	445 (52.5%)
Height (cm)	170 (9.03)
BMI (kg/m^2^)	27.2 (4.33)
Education level	
Low	136 (16.2%)
Intermediate	392 (46.7%)
High	312 (37.1%)
Early life livestock exposure	
No	343 (40.7%)
Childhood job at farm	200 (23.7%)
Farm childhood^a^	300 (35.6%)
Smoking status	
Never	386 (45.6%)
Current	40 (4.72%)
Former	421 (49.7%)
Atopy	247 (29.7%)
Self-reported COVID-19^b^	311 (36.7%)
Time between examinations (.00years)	7.41 (0.382)
Prebronchodilator lung function annual rate of change	
∆ FEV_1_ (ml/year)	−26.4 (32.3)
∆ FVC (ml/year)	−14.1 (37.8)
∆ FEV_1_/FVC × 100% (% per year)	−0.33% (0.49%)
∆ PEF (ml/second per year)	−61.2 (143)
∆ MMEF (ml/second per year)	−60.7 (68.8)
New onset airway obstruction (FEV_1_/FVC < 0.7)	
No	609 (71.9%)
Yes	106 (12.5%)
Baseline airway obstruction	132 (15.6%)
Livestock exposure	
Endotoxin (EU/m^3^)^c^ AM (SD)	0.246 (0.161)
Endotoxin (EU/m^3^)^c^ GM (GSD)	0.208 (1.80)
PM_10_ (µg/m^3^)^c^ AM (SD)	0.311 (0.177)
PM_10_ (µg/m^3^)^c^ GM (GSD)	0.265 (1.80)
∆ week prior avr. NH_3_ µg/m^3^ (T1–T0)	−2.46 (15.9)
N farms <1 km weighted to distance	0.0164 (0.0130)

Data are presented as arithmetic mean (SD) or n (%). Endotoxin and PM_10_ are presented as arithmetic and geometric mean (SD). Education levels: low, lower secondary school or less; intermediate, intermediate vocational education or upper secondary school; high, higher education or university.

a Growing up on a farm with or without performing farm jobs.

b Within 8 weeks of the follow-up examination.

c 2014–2015 annual average concentration at the home address by dispersion modeling.

### Exposure assessment

Long-term individual exposure to livestock emissions was defined as the baseline (2014–2015) annual-average livestock-related PM_10_ (µg/m^3^) and endotoxin (EU/m^3^) concentrations at the home address, predicted by previously developed advanced dispersion modeling.^22^ A Global Positioning System study performed in 2014–2016 found that VGO participants spend 87% of their time at home,^23^ showing that modeling based on the residential address is a good approximation of exposure. Briefly, to model the dispersion of air pollutants from a farm to the surroundings, a Gaussian plume model was used, which accounts for both vertical and horizontal dispersion.^22^ STACKS software was used for modeling, using the Netherlands New National Model as its basis.^24^ Model parameters used included farm-type specific PM emission factors, PM size distribution characteristics, and emission factors derived for endotoxin, as well as meteorological conditions and terrain roughness to estimate dispersion. To obtain annual-average concentrations at each residential address, we aggregated endotoxin emitted and dispersed from livestock farms in the vicinity (<10 km) and averaged over all 4-hour time windows for the years 2014–2015. Previous validation research showed dispersion modeling to be successful in quantifying endotoxin concentrations in ambient air at residential distances in the study area,^22^ moreover it better represented the range in exposure compared with Land Use Regression modeling, a stochastic modeling approach applied to endotoxin. Further details can be found in the Supplementary methods; https://links.lww.com/EE/A421.

### Data analysis

Data was analyzed in R Studio with R version 4.2.1.^25^ We first explored associations between residential exposure to livestock farming, emitted endotoxin, and PM_10_ with the annual rate of change in each lung function parameter between examinations. Since other studies of agricultural exposures and respiratory health measures have reported nonlinear associations,^8,11,22,26^ we employed penalized regression splines by means of the generalized additive model function from the mgcv-package (version 1.8-42) using the (default) “thin plate” basis with the number of knots set to 3.^27^ Additionally, associations between residential exposure to livestock farming emitted endotoxin and PM_10_ concentrations and the odds of self-reported asthma, wheeze and chronic cough, and new-onset airway obstruction were investigated using logistic regression. All models were adjusted for age, sex, smoking history (current, ever, and never), height, BMI, education level (low, intermediate, and high), atopy, and farm childhood as these were found to be relevant in earlier studies in the same population. Multicollinearity between covariates was checked using variance inflation factors. Details regarding post hoc analyses aimed to elucidate the role of farm childhood can be found in the Supplementary methods; https://links.lww.com/EE/A421.

## Results

### Study population

**Figure 2.**
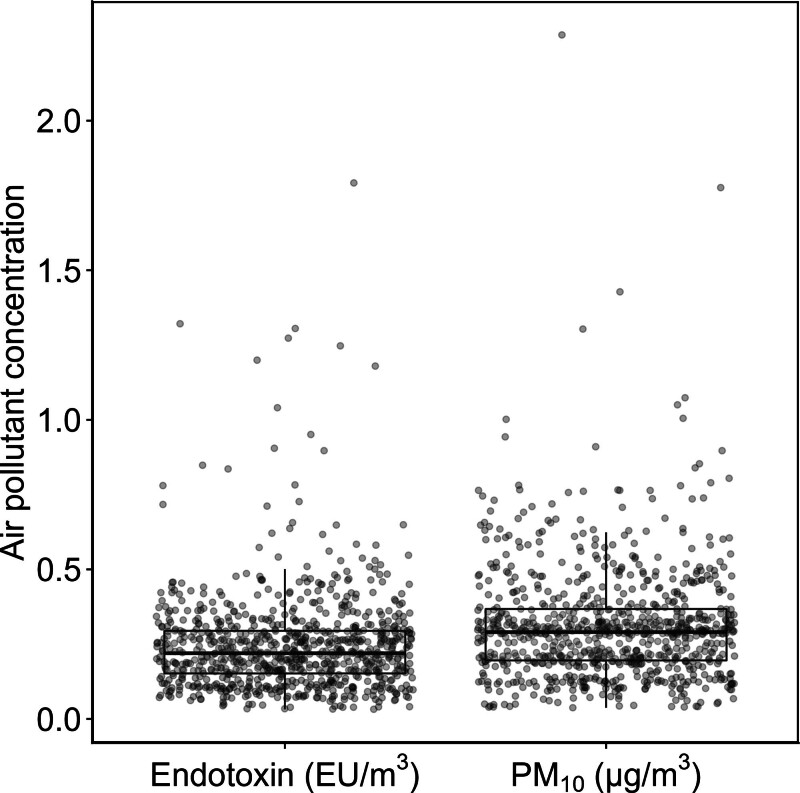
Baseline annual-average exposure to livestock farming emitted endotoxin at the home address estimated by dispersion modeling.

In total, 847 of 969 respondents who were visited for follow-up examination performed a reproducible lung function test. General characteristics of the study participants can be found in Table 1. More than half (55.6%) reported farm jobs during childhood, while over a third (35.6%) grew up on a livestock farm. The average decrease in prebronchodilator FEV_1_ was 26 ml/year (SD 32.3 ml/year), and the decrease in FVC was 14.1 ml/year (SD 37.8 ml/year). Descriptive statistics of spirometry results at both examinations, used to calculate the annual rate of change, can be found in Supplementary Table S1; https://links.lww.com/EE/A421. One out of eight (n = 106, 12.5%) participants, without airway obstruction (FEV_1_/FVC < 0.7) at baseline, had developed obstruction at the time of the follow-up examination. As shown in Supplementary Table S2; https://links.lww.com/EE/A421, asthma (self-reported doctor-diagnosed) was reported by 60 (7.1%) participants, chronic daily cough by 144 (17.2%), chronic daily phlegm by 120 (14.3%), and wheezing in the past year by 90 (10.8%). Almost all participants (97.8%) were vaccinated against COVID-19 when visited for follow-up (August 2021–July 2022, Supplementary Table S2; https://links.lww.com/EE/A421). The distribution of exposure to livestock farming emitted endotoxin and PM_10_ at participants’ home addresses can be found in Figure 2. Concentrations of both air pollutants were highly correlated (Pearson’s *r* = 0.79, *P* < 0.001). The relationship of early life farm exposure with participant characteristics is shown in Supplementary Table S3; https://links.lww.com/EE/A421. Participant characteristics of follow-up responders and nonresponders are shown in Supplementary Table S4; https://links.lww.com/EE/A421. Analyses on the association between the week prior NH_3_ exposure and baseline lung function show a similar negative effect in the responders and non-responders (Supplementary Table S5; https://links.lww.com/EE/A421). Associations between covariates, used to adjust regression splines, and baseline lung function are shown in Supplementary Table S6; https://links.lww.com/EE/A421.

### Lung function decline and livestock-related air pollutants

**Figure 3.**
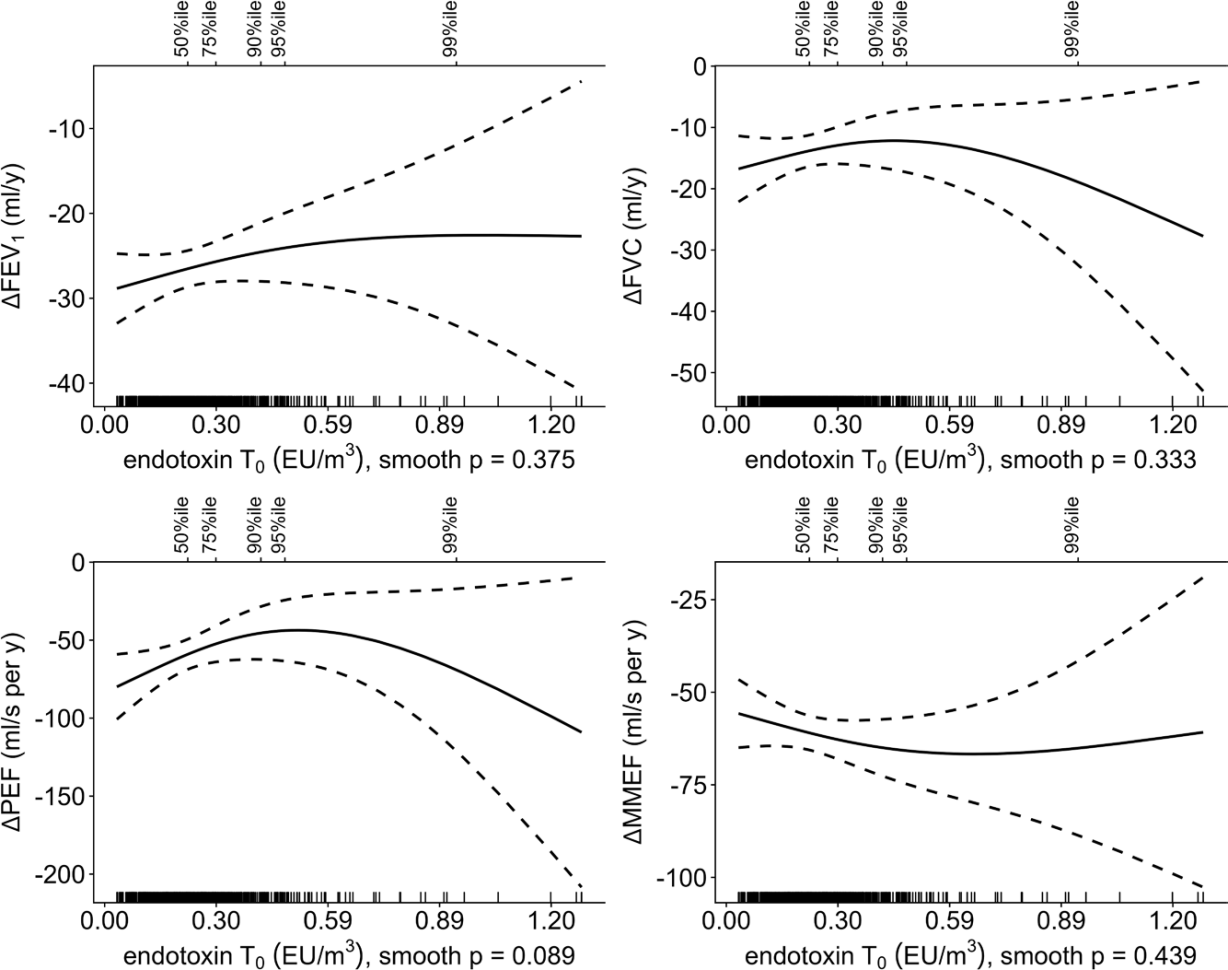
Splines of associations between annual rate of change in prebronchodilator lung function parameters and livestock-related endotoxin concentrations predicted by dispersion modeling. Dashed lines indicate 95% confidence intervals. Associations were adjusted for age, sex, height, BMI, education level, smoking history, atopy, and farm childhood. Rug plot shown on lower *x*-axis, percentiles shown on upper *x*-axis.

**Figure 4.**
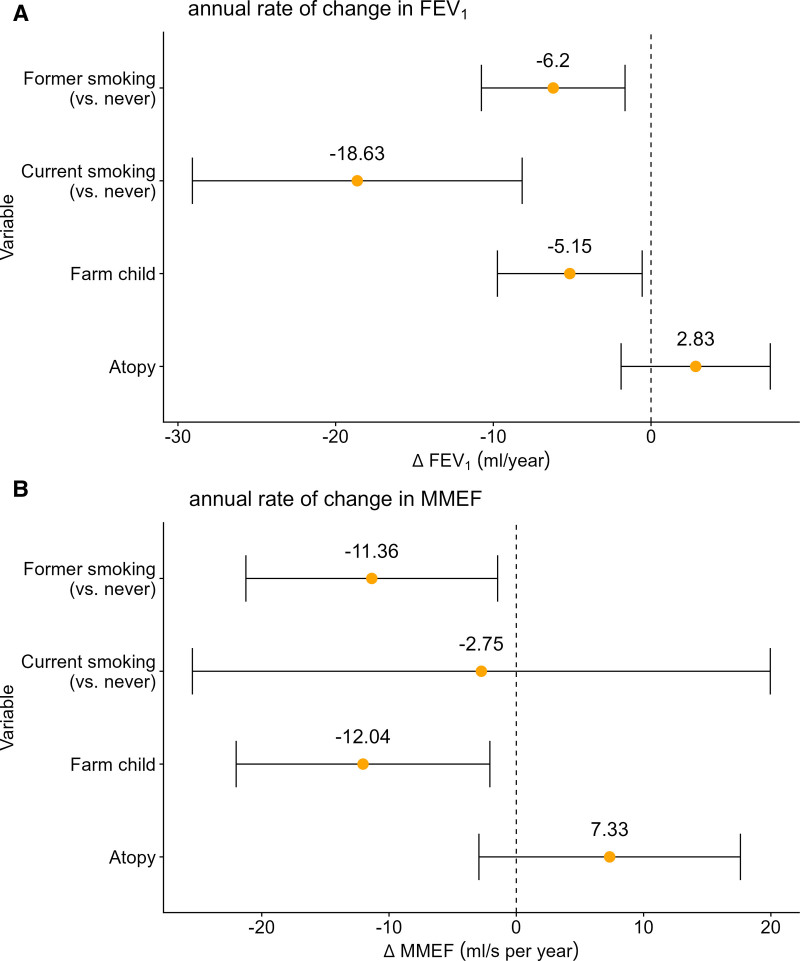
Generalized additive model covariates, used to adjust air pollutant regression splines, estimating the impact on (A) annual rate of change in prebronchodilator FEV_1_ and (B) annual rate of change in prebronchodilator MMEF over 7 years of follow-up.

Regression splines used to assess the relationship between livestock farming-emitted endotoxin and the annual rate of change in prebronchodilator lung function parameters can be found in Figure 3. No clear associations between FEV_1_, FVC, and PEF decline rate and residential exposure to endotoxin were observed. Also, no clear associations were identified for the annual rate of change in these lung function parameters with livestock-related PM_10_, see Supplementary Figure 1; https://links.lww.com/EE/A421. In addition, neither pollutant was associated with the rate of change in FEV_1_/FVC. The covariate model used to adjust the regression splines showed that living on a livestock farm in childhood was associated with accelerated annual decline in FEV_1_ (Figure 4, −5.15 ml/year, 95% confidence interval [CI] = −9.74, −0.56) and MMEF (−12.04 ml/second/year, 95% CI = −22.01, −2.08). A subgroup analysis in participants with and without atopy revealed that the association of farm childhood with accelerated FEV_1_ and MMEF decline was most pronounced in individuals without atopy (Supplementary Table S7; https://links.lww.com/EE/A421). Smoking was associated with accelerated annual decline in FEV_1_ (current and former smokers) and MMEF (only former smokers) compared with never smokers. Further details on the covariate model (without air pollution exposure) that was used to adjust the regression splines can be found in Supplementary Table S8; https://links.lww.com/EE/A421. Outcomes of additional sensitivity analyses were in line with the main findings, for instance adjusting for testing COVID-19 positive within 8 weeks of the home visit did not affect associations (see Supplementary Table S9; https://links.lww.com/EE/A421 for all results).

### Dichotomous respiratory health outcomes and livestock-related air pollutants

**Figure 5.**
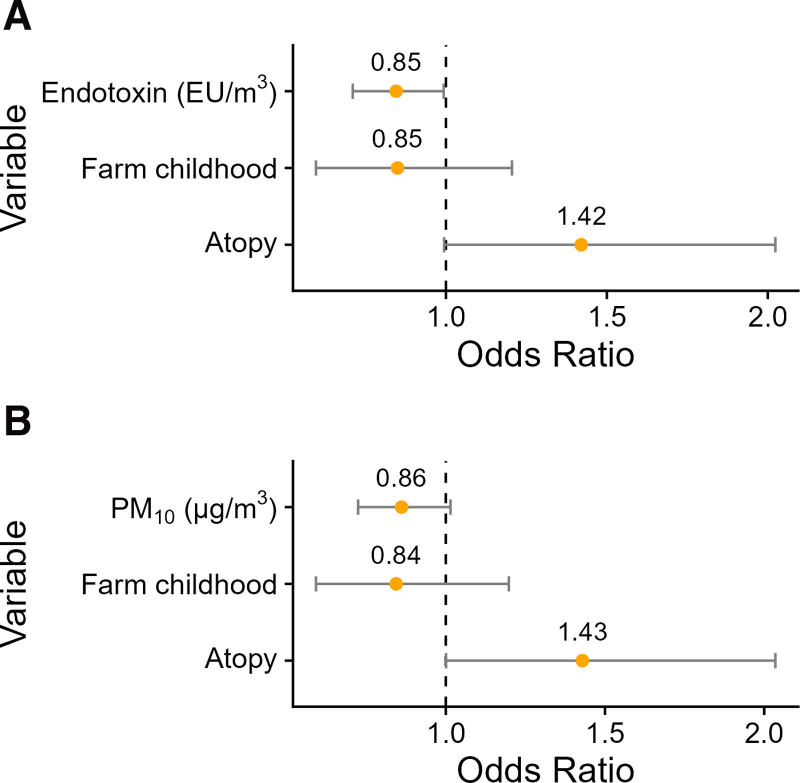
Logistic regression model associations between livestock-related air pollutants and new onset airway obstruction, defined as T0 FEV_1_/FVC > 0.7 and T1 FEV_1_/FVC < 0.7. A, depicts the endotoxin model, and (B) shows PM_10_.

We found that increased residential exposure to livestock farming emitted endotoxin was associated with lower odds of developing airway obstruction in the period between baseline and follow-up examinations (Figure 5, odds ratio = 0.85, 95% CI = 0.71, 0.99). A similar association was found for PM_10_ (odds ratio = 0.86, 95% CI = 0.73, 1.01). Associations between endotoxin, PM_10,_ and self-reported doctor-diagnosed asthma, chronic cough, chronic phlegm, and wheezing were close to the null and not statistically discernable (Supplementary Figure 2; https://links.lww.com/EE/A421). Both asthma and new-onset airway obstruction were less common in participants who grew up on a livestock farm compared with those who did not, but this difference was not statistically significant (*P* > 0.05).

## Discussion

This longitudinal study combines two prebronchodilator lung function measurements 7 years apart with dispersion modeled livestock emissions at the residential address, aiming to gain further insights into the relationship between long-term exposure to livestock emissions and lung function decline in adults. While cross-sectional studies provide convincing evidence that short-term and long-term residential exposure to livestock-related air pollution is associated with lower lung function, the current longitudinal study did not show evidence of a relationship between livestock-related air pollution and the rate of lung function decline in our nonfarming adult population.

Prior research on the effects of livestock-related air pollution on lung function, using cross-sectional data from the present cohort at baseline, has yielded noteworthy associations with lung function.^8,22^ Analyses on short-term exposure by Borlée et al.^8^ showed week-average NH_3_ concentrations to be negatively associated with FEV_1_ and MMEF. In the present study, focusing on the long-term effects of air pollutants on lung function decline, short-term air pollutant exposure before spirometry testing could have confounded our results. However, our sensitivity analysis, additionally adjusting for the difference in week-prior NH_3_ concentrations between examinations, indicated that the influence of short-term exposure on our results was negligible. The study by Borlée et al. also reported a lower MMEF with an increasing number of livestock farms within 1km of the home address. In the sensitivity analysis of the present study, no association with this exposure proxy was found (Supplementary Table S9; https://links.lww.com/EE/A421). The study by de Rooij et al.,^22^ incorporating more refined exposure modeling linked to the same baseline spirometry data, found that annual average endotoxin concentrations, but not PM_10_, tended to be associated with lower FVC. Investigating short-term exposures by copollutant modeling using the same baseline observations, FVC was found to be associated with week-average endotoxin exposure at the residential address in the week before spirometry testing.^16^ Also, FEV_1_ was negatively associated with week-average endotoxin exposure, but less consistent after copollutant adjustment. In addition, a cross-sectional study in a different Dutch cohort consisting of adolescents reported an association between long-term livestock-related PM_10_ exposure and lower FEV_1_.^15^ Our finding that endotoxin and PM_10_ exposure tend to be associated with decreased odds of developing airway obstruction is in line with two previous Dutch studies reporting inverse associations between livestock proximity and chronic obstructive pulmonary disease prevalence.^11,28^

Earlier literature, primarily focused on urban-related air pollution and lung function, revealed similar patterns; however, evidence from longitudinal studies on long-term effects is still modest.^4^ In 2015, the ESCAPE study reported a cross-sectional association between traffic-related air pollution and lower lung function in adults living in multiple cities across Europe.^2^ At the same time, no evidence for a relationship with longitudinal change in lung function was found. Later that year, however, a study from the United States reported an association of PM_2.5_ with lower FEV_1_ as well as greater annual FEV_1_ decline in adults.^6^ A more recent study in the United States investigated relationships between long-term ambient air pollution exposure with change in the occurrence of emphysema and lung function in adults.^29^ Higher baseline ozone (O_3_), nitrogen oxide (NO_x_), PM_2.5,_ and black carbon concentrations at the home address were associated with a greater increase in emphysema incidence, while only O_3_ concentrations at baseline and during follow-up were associated with accelerated FEV_1_ decline per 10 years.

Our results suggested that growing up on a livestock farm is associated with accelerated lung function decline (FEV_1_ and MMEF) later in life, in a nonfarming population, especially among nonatopic adults. This finding is of interest in light of the well-documented protective associations of childhood farm exposure with asthma and allergic sensitization that have been suggested to persist in adults.^30–33^ An explanation for these protective associations has been sought in the hygiene hypothesis or similar “old friends” hypothesis, revolving around microorganisms that coevolved with humans by embedding themselves (or their products) as regulatory inducers of immunologic pathways.^34^ Lack of exposure to these organisms (loss of “old friends”) is thought to decrease the immune system’s ability to modulate its response to allergens, causing it to overreact more often. In a study with 10,201 adults from 14 European countries, growing up on a farm was associated with higher FEV_1_ in women aged 26–54 years.^35^ It has been argued that this apparent beneficial effect of a farm childhood on lung function could be driven by the protective effect of childhood farm exposure on atopic sensitization.^36^ In our older study population, separate modeling for participants with and without atopy revealed associations between childhood farm exposure and lung function decline to be more pronounced in individuals without atopy. Analysis of baseline lung function, shown in Supplementary Table S6; https://links.lww.com/EE/A421, showed that those who grew up on a farm had a higher lung function when tested at a mean age of 54.7 years. So our study findings on the relationship between farm childhood and lung function decline between a mean age of 54.7 years and 63.3 years implies an accelerated decline in the later stages of life. Our results suggest that the initial beneficial effect of early life livestock exposure might disappear far later in life and could then potentially even have detrimental consequences. However, our single follow-up assessment limits conclusions on the extent to which the accelerated decline is explained by the initially higher baseline lung function and requires further follow-up in elderly populations. This bidirectionality in the health effects associated with early life microbial exposures, for example, the protective effect of endotoxin exposure on atopic conditions,^37,38^ and increased risk of nonatopic asthma,^39^ has also been described as “two sides of the same coin.”^40^

Considering the limitations of the present study, it would be premature to conclude that agricultural air pollutants have no impact on the natural rate of lung function decline in nearby residents. The lack of an observed association between livestock-related air pollution and lung function decline may, in part, be explained by COVID-19. Enrollment and subsequent fieldwork for the present study took place during the pandemic, which is likely to have decreased the number of respondents and selection towards relatively healthy individuals who felt comfortable with a home visit relatively soon after lockdown measures were lifted in the Netherlands. This is supported by the nonresponse analysis in the supplement (Supplementary Table S4; https://links.lww.com/EE/A421), showing that participants of follow-up examinations had a better baseline lung function, compared with participants who only underwent the baseline examination. This suggests that participants with worse baseline lung function tended to be lost to follow-up, which is partly explained by fewer smokers participating in follow-up. As a result, estimates for the lung function rate of change are presumed to be underestimated. Selective loss to follow-up (or attrition) is a known concern in longitudinal lung function studies, especially in older adults like the present cohort.^4^ Additionally, residential proximity to livestock farms has been associated with respiratory mortality,^41^ further highlighting potential selection bias towards individuals less sensitive to livestock exposure. However, the previously reported negative association between the week prior NH_3_ and baseline lung function was not found to differ between responders and nonresponders (Supplementary Table S5; https://links.lww.com/EE/A421).^8^ This shows that the effect of acute exposure to livestock emissions could also be detected in this subgroup with better lung function, suggesting that selection against individuals sensitive to livestock emissions was probably minor. This comparison, however, focusses on acute exposure and does not rule out selection due to spatial differences in long-term exposure. Lastly, because the rate of change in lung function was derived from two measurements, we could not investigate potential nonlinear trends of decline in lung function trajectories. Strengths of our study include the use of high-quality spirometry measurements. Apart from a software update, the exact same spirometers were used during baseline and follow-up, limiting potential bias introduced by equipment changes, a known issue of longitudinal lung function studies.^42^ Additionally, the dispersion modeled livestock farm emitted endotoxin, and PM_10_ concentrations at the home address offered a more specific and accurate residential exposure assessment compared with previously used crude exposure proxies.^8^

In conclusion, our longitudinal study did not find evidence for a relationship between air pollution from livestock farms and lung function decline in neighboring residents. Results suggest that early life exposure to livestock farming is associated with accelerated lung function decline in later life stages. Given that livestock industries are increasingly situated near densely populated areas across the globe, our study calls for future longitudinal studies with more observations over the life-course, assessing the impact of livestock-related air pollution on lung function trajectories in rural populations.

## Conflicts of interest statement

The authors declare that they have no conflicts of interest with regard to the content of this report.

## Supplementary Material



## References

[1] Milanzi EB Gehring U. Detrimental effects of air pollution on adult lung function. Eur Respir J. 2019;54:1901122. 31345990 10.1183/13993003.01122-2019

[2] Adam M Schikowski T Carsin AE. Adult lung function and long-term air pollution exposure. ESCAPE: a multicentre cohort study and meta-analysis. Eur Respir J. 2015;45:38–50. 25193994 10.1183/09031936.00130014 PMC4318659

[3] Garcia-Aymerich J Heras M Carsin AE. General population-based lung function trajectories over the life course: an accelerated cohort study. Lancet Respir Med. 2025;13:611–622. 40383131 10.1016/S2213-2600(25)00043-8 PMC12209707

[4] Gehring U Koppelman GH. Improvements in air quality: whose lungs benefit? Eur Respir J. 2019;53:1900365. 31023867 10.1183/13993003.00365-2019

[5] Lepeule J Litonjua AA Coull B. Long-term effects of traffic particles on lung function decline in the elderly. Am J Respir Crit Care Med. 2014;190:542–548. 25028775 10.1164/rccm.201402-0350OC PMC4214085

[6] Rice MB Ljungman PL Wilker EH. Long-term exposure to traffic emissions and fine particulate matter and lung function decline in the Framingham Heart Study. Am J Respir Crit Care Med. 2015;191:656–664. 25590631 10.1164/rccm.201410-1875OC PMC4384780

[7] Zhao T Markevych I Fuertes E. Impact of long-term exposure to ambient ozone on lung function over a course of 20 years (The ECRHS study): a prospective cohort study in adults. Lancet Reg Health Eur. 2023;34:100729. 37691742 10.1016/j.lanepe.2023.100729 PMC10482740

[8] Borlée F Yzermans CJ Aalders B. Air pollution from livestock farms is associated with airway obstruction in neighboring residents. Am J Respir Crit Care Med. 2017;196:1152–1161. 28489427 10.1164/rccm.201701-0021OC

[9] Schulze A Römmelt H Ehrenstein V. Effects on pulmonary health of neighboring residents of concentrated animal feeding operations: exposure assessed using optimized estimation technique. Arch Environ Occup Health. 2011;66:146–154. 21864103 10.1080/19338244.2010.539635

[10] Schinasi L Horton RA Guidry VT Wing S Marshall SW Morland KB. Air pollution, lung function, and physical symptoms in communities near concentrated swine feeding operations. Epidemiology. 2011;22:208–215. 21228696 10.1097/EDE.0b013e3182093c8b PMC5800517

[11] Borlée F Yzermans CJ van Dijk CE Heederik D Smit LAM. Increased respiratory symptoms in COPD patients living in the vicinity of livestock farms. Eur Respir J. 2015;46:1605–1614. 26250492 10.1183/13993003.00265-2015

[12] Loftus C Yost M Sampson P. Regional PM2.5 and asthma morbidity in an agricultural community: a panel study. Environ Res. 2015;136:505–512. 25460673 10.1016/j.envres.2014.10.030 PMC4425279

[13] Loftus C Yost M Sampson P. Ambient ammonia exposures in an agricultural community and pediatric asthma morbidity. Epidemiology. 2015;26:794–801. 26352250 10.1097/EDE.0000000000000368 PMC4587379

[14] van Kersen W Oldenwening M Aalders B. Acute respiratory effects of livestock-related air pollution in a panel of COPD patients. Environ Int. 2020;136:105426. 31881422 10.1016/j.envint.2019.105426

[15] Kiss P de Rooij MMT Koppelman GH. Residential exposure to livestock farms and lung function in adolescence - The PIAMA birth cohort study. Environ Res. 2023;219:115134. 36563981 10.1016/j.envres.2022.115134

[16] de Rooij MMT Erbrink HJ Smit LAM Wouters IM Hoek G Heederik DJJ. Short-term residential exposure to endotoxin emitted from livestock farms in relation to lung function in non-farming residents. Environ Res. 2024;243:117821. 38072102 10.1016/j.envres.2023.117821

[17] Cambra-López M Aarnink AJA Zhao Y Calvet S Torres AG. Airborne particulate matter from livestock production systems: a review of an air pollution problem. Environ Pollut. 2010;158:1–17. 19656601 10.1016/j.envpol.2009.07.011

[18] Sutton MA Howard CM Erisman JW, , eds. The European Nitrogen Assessment: Sources, Effects and Policy Perspectives. Cambridge University Press; 2011. doi:10.1017/CBO9780511976988

[19] Borlée F Yzermans CJ Krop E. Spirometry, questionnaire and electronic medical record based COPD in a population survey: comparing prevalence, level of agreement and associations with potential risk factors. PLoS One. 2017;12:e0171494. 28273094 10.1371/journal.pone.0171494 PMC5342260

[20] Quanjer PH Stanojevic S Cole TJ; ERS Global Lung Function Initiative. Multi-ethnic reference values for spirometry for the 3-95-yr age range: the global lung function 2012 equations. Eur Respir J. 2012;40:1324–1343. 22743675 10.1183/09031936.00080312 PMC3786581

[21] Graham BL Steenbruggen I Miller MR. Standardization of spirometry 2019 update. an official American Thoracic Society and European Respiratory Society technical statement. Am J Respir Crit Care Med. 2019;200:e70–e88. 31613151 10.1164/rccm.201908-1590ST PMC6794117

[22] de Rooij MMT Smit LAM Erbrink HJ. Endotoxin and particulate matter emitted by livestock farms and respiratory health effects in neighboring residents. Environ Int. 2019;132:105009. 31387023 10.1016/j.envint.2019.105009

[23] Klous G Smit LAM Borlée F. Mobility assessment of a rural population in the Netherlands using GPS measurements. Int J Health Geogr. 2017;16:30. 28793901 10.1186/s12942-017-0103-y PMC5551017

[24] Ham JV Duijm NJ Erbrink JJ. Revision of the Netherlands National Model for short range dispersion of air pollutants. Int J Environ Pollut. 1997;8:771–777.

[25] R Core Team. R: A Language and Environment for Statistical Computing. R Foundation for Statistical Computing; 2020. Available at: https://www.R-project.org/

[26] Borlée F Yzermans CJ Krop EJM. Residential proximity to livestock farms is associated with a lower prevalence of atopy. Occup Environ Med. 2018;75:453–460. 29712724 10.1136/oemed-2017-104769

[27] Wood SN. Stable and efficient multiple smoothing parameter estimation for generalized additive models. J Am Stat Assoc. 2004;99:673–686.

[28] Smit LAM Hooiveld M van der Sman-de Beer F. Air pollution from livestock farms, and asthma, allergic rhinitis and COPD among neighbouring residents. Occup Environ Med. 2014;71:134–140. 24142990 10.1136/oemed-2013-101485

[29] Wang M Aaron CP Madrigano J. Association between long-term exposure to ambient air pollution and change in quantitatively assessed emphysema and lung function. JAMA. 2019;322:546–556. 31408135 10.1001/jama.2019.10255 PMC6692674

[30] Douwes J Travier N Huang K. Lifelong farm exposure may strongly reduce the risk of asthma in adults. Allergy. 2007;62:1158–1165. 17845585 10.1111/j.1398-9995.2007.01490.x

[31] Elholm G Schlünssen V Doekes G. Become a farmer and avoid new allergic sensitization: adult farming exposures protect against new-onset atopic sensitization. J Allergy Clin Immunol. 2013;132:1239–1241. 23987793 10.1016/j.jaci.2013.07.003

[32] Radon K Schulze A Nowak D. Inverse association between farm animal contact and respiratory allergies in adulthood: protection, underreporting or selection? Allergy. 2006;61:443–446. 16512806 10.1111/j.1398-9995.2006.00995.x

[33] von Mutius E. The “hygiene hypothesis” and the lessons learnt from farm studies. Front Immunol. 2021;12:635522. 33868259 10.3389/fimmu.2021.635522 PMC8044987

[34] Rook GAW Lowry CA Raison CL. Microbial ‘Old Friends’, immunoregulation and stress resilience. Evol. Med. Public Health. 2013;2013:46–64. 24481186 10.1093/emph/eot004 PMC3868387

[35] Campbell B Raherison C Lodge CJ. The effects of growing up on a farm on adult lung function and allergic phenotypes: an international population-based study. Thorax. 2017;72:236–244. 27672121 10.1136/thoraxjnl-2015-208154

[36] Genuneit J von Mutius E. Do farm-grown lungs breathe better? Thorax. 2017;72:202–203. 27913768 10.1136/thoraxjnl-2016-209280

[37] Liu AH. Endotoxin exposure in allergy and asthma: reconciling a paradox. J Allergy Clin Immunol. 2002;109:379–392. 11897980 10.1067/mai.2002.122157

[38] Poole JA Romberger DJ. Immunological and inflammatory responses to organic dust in agriculture. Curr Opin Allergy Clin Immunol. 2012;12:126–132. 22306554 10.1097/ACI.0b013e3283511d0e PMC3292674

[39] Eduard W Douwes J Omenaas E Heederik D. Do farming exposures cause or prevent asthma? Results from a study of adult Norwegian farmers. Thorax. 2004;59:381–386. 15115863 10.1136/thx.2004.013326 PMC1747014

[40] Lee-Sarwar K. The farm-like effect: Rural exposures in early life, the microbiome, and asthma. J Allergy Clin Immunol. 2021;148:89–90. 33915125 10.1016/j.jaci.2021.04.020

[41] Simões M Janssen N Heederik DJJ Smit LAM Vermeulen R Huss A. Residential proximity to livestock animals and mortality from respiratory diseases in the Netherlands: a prospective census-based cohort study. Environ Int. 2022;161:107140. 35189407 10.1016/j.envint.2022.107140

[42] Gerbase MW Dupuis-Lozeron E Schindler C. Agreement between spirometers: a challenge in the follow-up of patients and populations? Respiration. 2013;85:505–514. 23485575 10.1159/000346649

