# Supplementary material for: Air pollution from livestock farms and lung function decline in neighboring residents over 7 years
Source: Environ Epidemiol. 2026 Apr 15;10(3):e479. doi: 10.1097/EE9.0000000000000479 (PMC13086416; doi:10.1097/EE9.0000000000000479)
Supplement: Supplementary file 1 [file ee9-10-e479-s001.pdf]

Air pollution from livestock farms and lung function decline in neighboring residents over 7 years

Warner van Kersen, Myrna M.T. de Rooij, Bernadette Aalders, Kaitlin Prins, Floor Borlée, Joris C. Yzermans, Joke W.B. van der Giessen, Dick Heederik, Mary B. Rice, Lidwien A.M. Smit

SUPPLEMENT

Supplementary methods

Questionnaire

1 What is your sex?

❍ Male ❍ Female

2 What is your birthdate?

|__|__| |__|__| |__|__|__|__|

Day month year

3 Have you had wheezing or whistling in your chest at any time in the last 12 months?

❍ Yes ❍No

6 Have you ever had asthma?

❍ Yes ❍No (***if no**, continue with question 8*)

If yes, ...

7 Was the asthma confirmed by a doctor?

❍ Yes ❍ No

8 Do you cough nearly daily, for as long as three months per year?

❍ Yes ❍ No

9 Do you produce phlegm nearly daily, for as long as three months per year?

❍ Yes ❍ No

10 Have you had pneumonia in the past 3 years?

❍ Yes ❍ No

10 As a child (until 18 years old), did you live on a farm with animals?

❍ Yes ❍ No

11 As a child (up to the age of 18), did you carry out one or more of the following activities on a farm? (multiple answers possible)

❍ Animal care with intensive animal contact

❍ Working with manure

- ❍ Working with straw / hay / grass silage / animal feed
- ❍ Crop care
- ❍ None of the above

12 Have you visited a farm in the past 12 months?
(*multiple answers possible)*

- ❑ Yes, visiting family
- ❑ Yes, for work
- ❑ Yes, to buy produce (e.g. vegetables, fruit, eggs or meat)
- ❑ Yes, a petting zoo
- ❑ Yes, for another reason
- ❑ No

Spirometry

Particulate matter air pollution, as well as airborne endotoxin exposure, is associated with decreases in parameters of both large airways and small airways [1, 2]. Large airway function is assessed with parameters: Forced Expiratory Volume in 1 second (FEV1), Forced Vital Capacity (FVC) and the ratio between these two (FEV1/FVC), small airway function is assessed with Peak Expiratory Flow (PEF) and Maximum Mid-Expiratory Flow (MMEF). During both examinations pre-bronchodilator (BD) spirometry was conducted according to ERS/ATS standards [3]. Pre-BD spirometry measures the lung function of the participant without any effect of lung medication. Participants stopped using inhalers and oral lung medication 4 and 8 hours prior to the examination. The EasyOne Spirometer (NDD Medical Technologies, Inc.) was used which measures flow and volume by ultra-sound transit time.

To increase the quality of the spirometry data, we attempted to obtain four acceptable spirograms per participant. In addition, an expert reviewed the quality of all lung function curves in the NDD software. In the quality review process, the three best curves were ranked manually based on predefined ERS criteria [3]. Additional exclusions were made after inspecting scatterplots of FEV_1 and FVC at baseline and follow-up in combination with questionnaire data. One participant with standout lower FEV_1 and FVC at follow-up (compared to baseline) was excluded due to bruised ribs during follow-up examination. In addition, 5 participants were excluded as the data suggested that the follow-up examination was not performed by the same individual as at baseline (e.g., different birthdate with an abnormal increase in lung function). Only data from participants with a spirometry quality grade C (at least two reproducible curves within 200ml) or higher were used in the downstream analysis.

Secondary respiratory health outcomes

New onset airway obstruction was defined as FEV1/FVC>0.7 at T_0 and FEV1/FVC<0.7 at T_1, based on the global initiative for chronic lung disease (GOLD stage 1 and higher) [29]. Self-reported doctor diagnosed asthma, chronic cough, chronic phlegm and wheezing were collected via (ECRHS-III based) questionnaire as dichotomous respiratory health measures (supplementary methods). Additionally, atopy was defined as one or both of 1) elevated levels of specific IgE antibodies (>0.35 U/ml) to at least one common allergen (cat, dog, grass, house dust mite) and 2) total IgE exceeding 100 IU/ml assessed by ELISA in serum samples collected at T0 [22, 30].

Dispersion modelling

Residential exposure was estimated by applying dispersion modelling to individual barns within 10km of each residential address. Endotoxin emission and dispersion was modelled by including farm-type specific endotoxin content per PM size fraction. Unrealistically high values in residential exposures, resulting from an erroneous overlap in geocoordinates of home addresses and farms, were winsorized to the 99.5 percentile in the downstream analysis. Additionally, as an exposure proxy, distance of the home address to the nearest farm was included which was based on provincial livestock data of 2015 (https://veehouderijen.igoview.nl). Lastly, average ambient NH_3 concentrations (μg/m^3) in the week prior to spirometry at T_0 and T_1 were calculated as a proxy for short term exposure to livestock emissions. This was done using hourly observations in the study area, obtained from the national air quality monitoring network (https://www.luchtmeetnet.nl).

Early life and other farm exposures

Information on early life and other farm exposures were extracted from questionnaire data from closed questions (yes/no) about having lived on a farm childhood, performing farm jobs during childhood, and whether farms were visited in the past year.

Secondary data analysis

The role of atopic predisposition on the association between agricultural exposures and lung function, was evaluated in a subgroup analysis. To this end, we analyzed exposure-response relationships among individuals with and without atopy separately. To distinguish between childhood and current livestock exposures, we ran secondary models additionally adjusting for distance to nearest farm (m), the number of farms within a 1km buffer around the home address weighted to distance and farm visits (yes/no). As spirometry measurements were performed during the COVID-19 pandemic in 2021 and 2022, and COVID-19 infection could have an impact on lung function, we also ran models additionally adjusting for self-reported COVID-19 within 8 weeks of the measurement (yes/no). We investigated whether variability introduced by short-term effects of livestock air pollution exposure at both timepoints influenced our results. To achieve this, we ran models additionally adjusting for the difference between follow-up and baseline NH_3 concentration in the weeks prior to both spirometry measurements. Differences in baseline characteristics between participants who did or did not partake in the follow-up examination were

explored in a linear model. The potential impact of selection bias was further explored by assessing whether associations between NH_3 exposure and lung function were similar in participants who did and did not respond to the follow-up invitation. To this end we ran a linear model with an interaction term for baseline week prior NH_3 concentrations and follow-up participation.

Supplementary results

Childhood farm exposure, participant characteristics and follow-up response. Individuals with a farm childhood typically lived closer to livestock farms compared to those without ($p<0.001$, Supplementary table S3). Additionally, those with a farm childhood were more likely to report farm visits in the past year (60.7% vs 45.2%, $p<0.001$). Performing farm jobs during childhood was highly prevalent among those growing up on a farm (89.3% vs 36.8%, $p<0.001$). However, even in those who did not live on a farm during childhood, 36.8% reported working on a farm during childhood. Characteristics of participants who agreed to the follow-up examination, compared to those who did not, can be found in supplementary table S4. Compared to non-responders, the population followed-up had a higher percentage of males (49.6% vs 42.6%) and tended to be higher educated (35.8% vs 26.7%). Baseline lung function was slightly better in respondents, with a higher %-predicted FEV_1 (100% vs 98.3%), FVC (103% vs 102%) and MMEF (96.6% vs 92.5%). A linear model for baseline spirometry (expressed in %-predicted for comparison with Borlée et al. [4]) with an interaction term for average NH_3 concentration in the week prior to lung function testing and response to follow-up, found no difference in the association between NH_3 exposure and lung function between responders and non-responders (Supplementary table S5).

Sensitivity analysis

Additionally adjusting regression splines for distance to the nearest farm, N farms <1km weighted to distance, farm visits, performing farm jobs during childhood, COVID-19 within 8 weeks of the follow-up examination or change in week prior NH_3 exposure, did not significantly alter the observed relationships between farm childhood and lung function (supplementary table S9).

References

1. Farokhi A, Heederik D, Smit LAM. Respiratory health effects of exposure to low levels of airborne endotoxin – a systematic review. *Environ. Health* 2018; 17: 14.

2. van der Wiel E, ten Hacken NHT, Postma DS, van den Berge M. Small-airways dysfunction associates with respiratory symptoms and clinical features of asthma: a systematic review. *J. Allergy Clin. Immunol.* 2013; 131: 646–657.

3. Graham BL, Steenbruggen I, Miller MR, Barjaktarevic IZ, Cooper BG, Hall GL, Hallstrand TS, Kaminsky DA, McCarthy K, McCormack MC, Oropez CE, Rosenfeld M, Stanojevic S, Swanney MP, Thompson BR. Standardization of Spirometry 2019 Update. An Official American Thoracic Society and European Respiratory Society Technical Statement. *Am. J. Respir. Crit. Care Med.* American Thoracic Society - AJRCCM; 2019; 200: e70–e88.

4. Borlée F, Yzermans CJ, Aalders B, Rooijackers J, Krop E, Maassen CBM, Schellevis F, Brunekreef B, Heederik D, Smit LAM. Air Pollution from Livestock Farms Is Associated with Airway Obstruction in Neighboring Residents. *Am. J. Respir. Crit. Care Med.* 2017; 196: 1152–1161.

Supplementary Tables and Figures

Supplementary Table S1: pre-bronchodilator lung function parameters in 2014-2015 (T_0) and 2021-2022 (T_1) measured during the VGO study

	T_0 (N=847)	T_1 (N=847)	p-value*
**FEV_1 (L)**	3.21 (0.74)	3.01 (0.75)	<0.001
**FVC (L)**	4.22 (0.99)	4.12 (0.98)	0.02
**FEV_1/FVC %**	76.10 (6.51)	73.7 (7.32)	<0.001
**PEF (L/sec)**	8.51 (2.04)	8.05 (2.11)	<0.001
**MMEF (L/sec)**	2.75 (1.06)	2.30 (1.02)	<0.001

Data are presented as mean (SD). * t-test

Supplementary Table S2: dichotomous respiratory health outcomes of VGO 7-year follow-up study

	Overall (N=847)
**Self-reported doctor diagnosed asthma**	60 (7.08%)
**Daily cough >3m last year**	144 (17.2%)
**Daily phlegm >3m last year**	120 (14.3%)
**Wheezing last year**	90 (10.8%)
**Self-reported COVID-19**	
no	536 (63.3%)
yes, tested	253 (29.9%)
yes, suspected	58 (6.85%)
**COVID-19 <8wks of visit**	72 (8.63%)
**COVID-19 vaccinated prior to study**	818 (97.8%)

Data are presented as n (%).

Supplementary Table S3: VGO follow-up study participant characteristics stratified by farm childhood

Living on a farm in childhood	no (N=543)	yes (N=300)	p-value*
**Distance to nearest farm (m)**	448 (253)	380 (241)	<0.001
**Farm visit in past year**	242 (45.2%)	179 (60.7%)	<0.001
**Multiple reasons for farm visit**			
No, did not visit	293 (54.8%)	116 (39.3%)	<0.001
No, single reason	195 (36.4%)	135 (45.8%)	
yes	47 (8.79%)	44 (14.9%)	
**Childhood farm job**	200 (36.8%)	268 (89.3%)	<0.001
**Atopy**	183 (34.0%)	64 (22.0%)	<0.001
**2014-15 pre-bronchodilator lung function**			
FEV_1 (L)	3.24 (0.747)	3.16 (0.737)	0.14
FVC (L)	4.28 (0.986)	4.13 (0.873)	0.04
FEV_1/FVC %	76.0 (6.75)	76.4 (6.09)	0.44
PEF (L/sec)	8.56 (2.06)	8.41 (2.01)	0.32
MMEF (L/sec)	2.76 (1.04)	2.76 (1.09)	0.99
**2021-22 pre-bronchodilator lung function**			
FEV_1 (L)	3.06 (0.751)	2.93 (0.738	0.02
FVC (L)	4.18 (1.01)	4.00 (0.915)	0.01
FEV_1/FVC %	73.8 (7.40)	73.6 (7.22)	0.74
PEF (L/sec)	8.13 (2.07)	7.90 (2.17)	0.13
MMEF (L/sec)	2.34 (1.02)	2.23 (1.02)	0.13

Data are presented as mean (SD) or n (%). * t-test or chi^2

Supplementary Table S4: VGO participant baseline characteristics, comparing follow-up responders and non-responders

	Non-responders (N=1525)	responders (N=969)	p-value*
**Age at baseline**	56.5 (11.7)	56.4 (9.99)	0.946
**female**	875 (57.4%)	488 (50.4%)	<0.001
**Height (cm)**	170 (0.90)	172 (0.90)	<0.001
**BMI (kg/m^2)**	27.2 (4.45)	26.9 (3.94)	0.140
**Education level**			
low	439 (29.2%)	168 (17.5%)	<0.001
intermediate	662 (44.0%)	450 (46.8%)	
high	402 (26.7%)	344 (35.8%)	
**Early-life livestock exposure**			
no	642 (42.7%)	391 (40.5%)	0.295
farm job	370 (24.6%)	229 (23.7%)	
farm childhood	492 (32.7%)	345 (35.8%)	
**Atopy**	447 (30.0%)	280 (29.4%)	0.779
**Smoking**			
non	629 (41.2%)	430 (44.4%)	0.023
former	735 (48.2%)	467 (48.2%)	
current	161 (10.6%)	72 (7.43%)	
**Pre-bronchodilator lung function**			
percent predicted FEV_1 †	98.3 (16.0)	100 (14.5)	0.001
percent predicted FVC†	102 (13.4)	103 (12.8)	0.040
percent predicted MMEF†	92.5 (33.5)	96.8 (32.8)	0.002
FEV_1/FVC (%)	95.4 (9.26)	96.5 (8.11)	0.002
**Livestock exposure**			
Endotoxin (EU/m3)‡	0.247 (0.169)	0.247 (0.157)	0.898
PM_{10} (µg/m3)‡	0.302 (0.187)	0.311 (0.176)	0.214
Distance to nearest farm (m)	446 (273)	427 (252)	0.065

Data are presented as mean (SD) or n (%). Education levels: low = lower secondary school or less; intermediate = intermediate vocational education or upper secondary school; high = higher education or university.† percentage of predicted value calculated conform GLI 2012 reference equations. ‡ Baseline annual average concentration at the home address by dispersion modeling. * t-test or chi^2.

Supplementary Table S5: linear model results for percentage predicted baseline pre-bronchodilator lung function with interaction for week average NH_3 and follow-up examination

	FEV_1 % predicted		FVC % predicted		FEV_1/FVC %		MMEF % predicted	
	β	95% CI	β	95% CI	β	95% CI	β	95% CI
**Week prior avr. NH_3** (µg/m3)	-6.52	-12.88, -0.16	-0.75	-2.44, 0.94	-2.88	-5.45, -0.31	-3.8	-6.79, -0.81
**Smoking history**								
current vs never	-22.63	-27.48, -17.78	-7.11	-8.4, -5.82	-1.77	-3.73, 0.19	-8.61	-10.9, -6.33
former vs never	-2.13	-4.99, 0.73	-1.81	-2.57, -1.05	1.38	0.22, 2.53	-0.34	-1.68, 1.01
**Born in study area**	1.19	-2.08, 4.45	-0.6	-1.47, 0.27	2.45	1.13, 3.77	1.82	0.29, 3.36
**Farm childhood**	6.86	3.93, 9.8	0.82	0.04, 1.6	1.88	0.69, 3.06	2.68	1.3, 4.06
**Follow-up examination**	0.91	-6.03, 7.86	0.99	-0.86, 2.83	-0.39	-3.19, 2.42	0.22	-3.05, 3.49
**Week average NH_3 x Follow-up examination**	3.74	-6.2, 13.67	-0.32	-2.96, 2.32	2.51	-1.51, 6.52	2.51	-2.17, 7.19

All variables were mutually adjusted. % predicted values were calculated conform 2012 GLI reference equations.

Supplementary Table S6: Generalized additive model associations between covariates and baseline pre-bronchodilator lung function

	**FEV_1 (ml)**		**FVC (ml)**		**FEV_1/FVC (%)**		**PEF (ml/s)**		**MMEF (ml/s)**	
	β	95%CI	β	95%CI	β	95%CI	β	95%CI	β	95%CI
**Age (per 10y)**	-251.58	-288.84, -214.31	-209.29	-251.18, -167.41	-2.14	-2.63, -1.65	-322.18	-431.56, -212.79	-459.91	-532.8, -387.02
**Female**	-528.59	-622.35, -434.83	-711.31	-816.68, -605.94	0.32	-0.91, 1.55	-2278.73	-2553.92, -2003.54	-427.83	-611.2, -244.46
**Height (cm)**	35.37	30.25, 40.49	52.89	47.14, 58.65	-0.10	-0.17, -0.03	56.95	41.92, 71.97	19.95	9.94, 29.96
**BMI (kg/m^2)**	-9.18	-16.81, -1.56	-17.62	-26.19, -9.05	0.07	-0.03, 0.17	4.43	-17.96, 26.82	4.07	-10.85, 18.99
**Education level (vs low)**										
medium	13.49	-80.54, 107.53	-10.26	-115.93, 95.41	0.40	-0.83, 1.63	116.82	-159.16, 392.81	39.21	-144.69, 223.11
high	31.96	-66.60, 130.51	42.72	-68.04, 153.48	0.14	-1.15, 1.43	259.55	-29.71, 548.81	-38.77	-231.51, 153.98
**Smoking history (vs never)**										
current	-190.86	-346.06, -35.67	-16.88	-191.29, 157.53	-4.13	-6.16, -2.10	-413.01	-868.51, 42.49	-574.79	-878.31, -271.26
former	-8.13	-75.80, 59.54	31.49	-44.56, 107.55	-0.68	-1.57, 0.21	117.29	-81.33, 315.92	-47.69	-180.04, 84.66
**Farm childhood**	40.06	-28.16, 108.27	-21.22	-97.88, 55.44	0.94	0.05, 1.83	23.28	-176.92, 223.48	169.25	35.84, 302.65
**Atopy**	-13.55	-83.78, 56.68	53.45	-25.48, 132.38	-1.30	-2.22, -0.38	-8.23	-214.37, 197.9	-95.79	-233.15, 41.57

All variables were mutually adjusted.

Supplementary Table S7: generalized additive model covariate results for smoking and childhood farm exposure with pre-bronchodilator lung function in participants with and without atopy

	ΔFEV1 (ml/y)		ΔFVC (ml/y)		ΔFEV1/FVC (%/y)		ΔPEF (ml/s per year)		ΔMMEF (ml/s per year)	
	β	95%CI	β	95%CI	β	95%CI	β	95%CI	β	95%CI
**Smoking current vs never**										
atopic	-39.00	-57.91, -20.09	-54.58	-77.10, -32.05	0.04	-0.24, 0.32	-77.14	-159.60, 5.33	2.79	-36.28, 41.86
non-atopic	-11.18	-23.51, 1.15	-9.72	-24.34, 4.90	-0.10	-0.30, 0.09	-89.86	-146.11, -33.62	-7.07	-34.40, 20.26
**Smoking former vs never**										
atopic	-12.16	-20.76, -3.56	-16.74	-26.99, -6.49	-0.02	-0.15, 0.10	-31.13	-68.64, 6.38	-11.10	-28.87, 6.67
non-atopic	-4.39	-9.66, 0.87	-2.60	-8.84, 3.64	-0.06	-0.14, 0.03	12.23	-11.78, 36.25	-13.03	-24.70, -1.36
**farm childhood**										
atopic	-0.66	-11.58, 10.25	2.64	-10.36, 15.64	0.03	-0.14, 0.19	20.57	-27.04, 68.18	-15.89	-38.45, 6.66
non-atopic	-6.58	-12.61, -0.54	-1.97	-9.13, 5.18	-0.13	-0.22, -0.03	-18.50	-46.04, 9.03	-14.23	-27.61, -0.85
**Childhood farm job**										
atopic	0.29	-9.24, 9.82	-2.75	-14.10, 8.60	-0.02	-0.16, 0.12	-27.70	-69.26, 13.86	4.20	-15.49, 23.88
non-atopic	1.19	-4.82, 7.19	-3.54	-10.66, 3.58	0.12	0.03, 0.22	20.57	-6.81, 47.96	7.29	-6.01, 20.60

Models were adjusted for age, sex, height, BMI and education level. If relevant, models were additionally adjusted for smoking history, farm childhood and childhood farm job.

Supplementary Table S8: Generalized additive model associations between covariates and pre-bronchodilator lung function rate of change over seven years of follow-up

	**ΔFEV1 (ml/y)**		**ΔFVC (ml/y)**		**ΔFEV1/FVC (%/y)**		**ΔPEF (ml/s per year)**		**ΔMMEF (ml/s per year)**	
	β	95% CI	β	95% CI	β	95% CI	β	95% CI	β	p value
**Age (per 10y)**	-3.95	-6.46, -1.44	-4.90	-7.85, -1.94	-0.02	-0.06, 0.02	-23.31	-34.7, -11.91	-2.58	-8.03, 2.87
**Female**	1.92	-4.39, 8.23	-5.64	-13.07, 1.79	0.06	-0.03, 0.16	-13.33	-41.98, 15.33	22.68	8.98, 36.38
**Height (cm)**	-0.21	-0.56, 0.13	-0.10	-0.5, 0.31	0.001	0.00, 0.01	-0.84	-2.4, 0.73	0.19	-0.56, 0.93
**BMI (kg/m²)**	-0.59	-1.1, -0.07	-1.26	-1.87, -0.66	0.01	0.00, 0.02	-2.44	-4.77, -0.11	0.21	-0.9, 1.32
**Education level**										
intermediate (vs low)	3.81	-2.52, 10.15	3.46	-4.00, 10.91	0.08	-0.02, 0.17	22.70	-6.06, 51.46	1.57	-12.18, 15.33
high (vs low)	1.38	-5.25, 8.01	4.67	-3.14, 12.47	0.01	-0.09, 0.12	7.34	-22.78, 37.45	-4.18	-18.58, 10.22
**Smoking history**										
Current (vs never)	-18.63	-29.08, -8.17	-22.67	-34.98, -10.36	-0.05	-0.21, 0.11	-81.84	-129.32, -34.37	-2.75	-25.45, 19.95
Former (vs never)	-6.20	-10.76, -1.65	-6.65	-12.01, -1.29	-0.05	-0.12, 0.03	4.06	-16.61, 24.74	-11.36	-21.24, -1.47
**Farm childhood**	-5.15	-9.74, -0.56	-2.95	-8.35, 2.45	-0.04	-0.11, 0.03	-8.24	-29.08, 12.6	-12.04	-22.01, -2.08
**Atopy**	2.83	-1.90, 7.56	1.24	-4.33, 6.81	0.02	-0.05, 0.09	4.43	-17.05, 25.91	7.33	-2.94, 17.61

All variables were mutually adjusted.

Supplementary Table S9: results of generalized additive model for farm childhood, additionally adjusted for distance to the nearest farm, N farms <1km weighted to distance, farm visits, childhood farm jobs, COVID-19 within 8 weeks of follow-up examination and change in week prior average NH_3 concentration.

	**ΔFEV1 (ml/y)**		**ΔFVC (ml/y)**		**ΔFEV1/FVC (%/y)**		**ΔPEF (ml/s per year)**		**ΔMMEF (ml/s per year)**	
	β	95%CI	β	95%CI	β	95%CI	β	95%CI	β	95%CI
**Base model**										
Farm childhood	-4.66	-9.21, -0.11	-2.75	-8.16, 2.66	-0.04	-0.11, 0.03	-5.05	-25.46, 15.37	-10.69	-20.50, -0.88
**Distance to farm**										
Farm childhood	-4.48	-9.08, 0.11	-2.00	-7.45, 3.45	-0.05	-0.12, 0.02	-5.75	-26.38, 14.88	-10.59	-20.5, -0.67
log distance to the nearest farm (m)	0.85	-2.23, 3.93	3.57	-0.08, 7.22	-0.03	-0.08, 0.01	-3.33	-17.15, 10.50	0.49	-6.16, 7.13
**N farms <1km weighted to distance**										
Farm childhood	-4.83	-9.44, -0.24	-2.54	-8.02, 2.94	-0.05	-0.12, 0.02	-6.18	-26.88, 14.52	-10.44	-20.39, -0.49
N farms <1km weigh to distance	36.27	-132.40,204.93	-46.86	-247.23, 153.52	1.60	-1.02, 4.21	254.04	-502.58, 1010.66	-56.20	-419.89, 307.48
**Farm visits**										
Farm childhood	-4.76	-9.40, -0.13	-2.89	-8.40, 2.62	-0.04	-0.12, 0.03	-6.98	-27.84, 13.89	-9.49	-19.52, 0.53
farm visits	1.90	-2.44, 6.24	1.12	-4.04, 6.29	0.01	-0.06, 0.08	9.59	-9.97, 29.15	2.45	-6.94, 11.85
**Farm jobs during childhood**										
Farm childhood	-5.11	-10.43, 0.22	-1.01	-7.33, 5.30	-0.08	-0.17, -0.0004	-8.04	-31.91, 15.83	-14.15	-25.61, -2.68
Farm job during childhood	0.82	-4.28, 5.91	-3.21	-9.27, 2.84	0.07	-0.005, 0.15	5.53	-17.34, 28.40	6.40	-4.59, 17.38
**COVID-19 prior to examination**										
Farm childhood	-4.48	-9.09, 0.14	-2.85	-8.34, 2.63	-0.04	-0.05, -0.04	-2.26	-22.87, 18.36	-9.84	-19.75, 0.06
COVID-19 within 8 wks of examination	-1.42	-9.10, 6.27	-6.33	-15.47, 2.81	0.06	0.07, 0.07	16.11	-18.23, 50.46	3.87	-12.64, 20.37
**Change in week prior avr. NH_3**										
Farm childhood	-4.68	-9.24, -0.13	-2.71	-8.12, 2.70	-0.04	-0.11, 0.03	-4.69	-25.11, 15.73	-10.89	-2.96, 17.25
Change in week prior avr. NH_3 (μg/m3)	-0.02	-0.15, 0.11	0.04	-0.12, 0.20	-0.001	-0.003, 0.001	0.36	-0.23, 0.96	-0.21	-0.49, 0.08

All variables were mutually adjusted. Models were additionally adjusted for age, gender, height, BMI, education level, smoking history and atopy

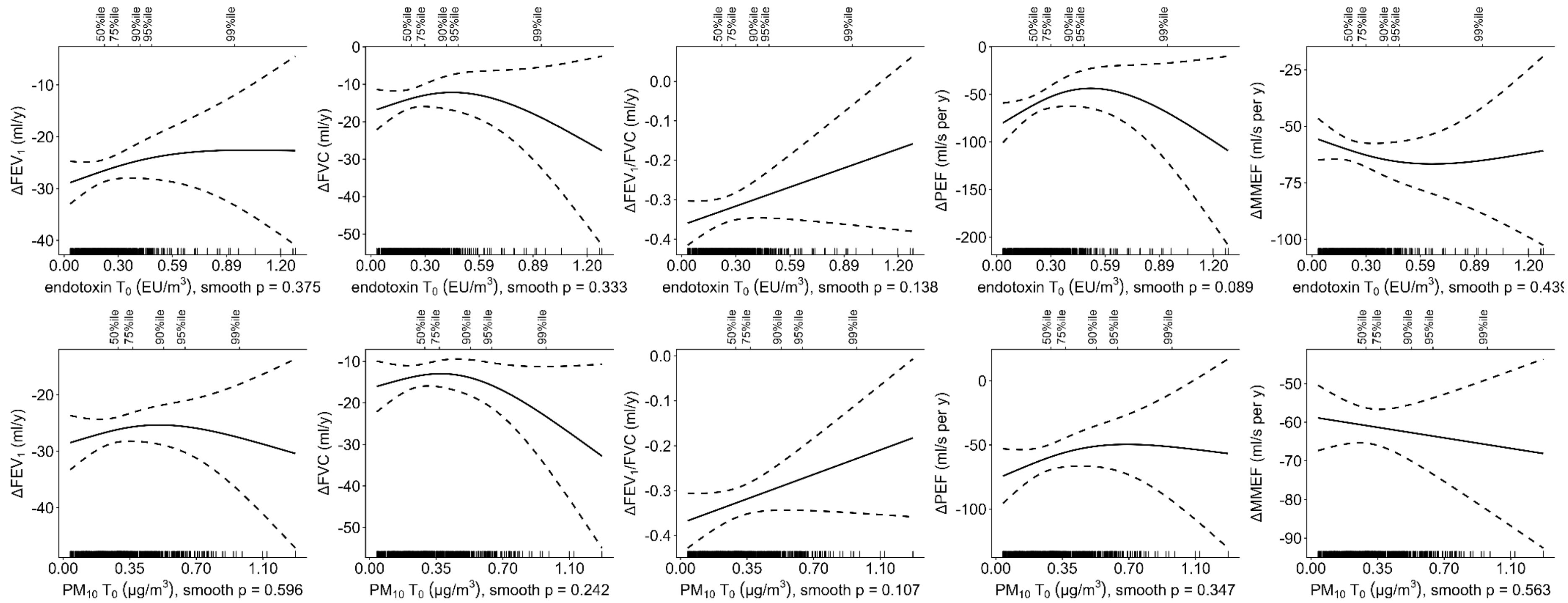

Supplementary figure 1: Splines of associations between annual rate of change in lung function parameters and livestock-related endotoxin and PM_{10} concentrations predicted by dispersion modelling.

Note. Number of knots was set to 3. Dashed lines indicate 95% confidence intervals. Associations were adjusted for age, sex, height, BMI, education level, smoking history, atopy, farm childhood. Rug plot shown on lower x-axis, percentiles shown on upper x-axis. Predicted livestock emitted air pollutant concentrations at residential addresses were winsorized to 99.5 percentile

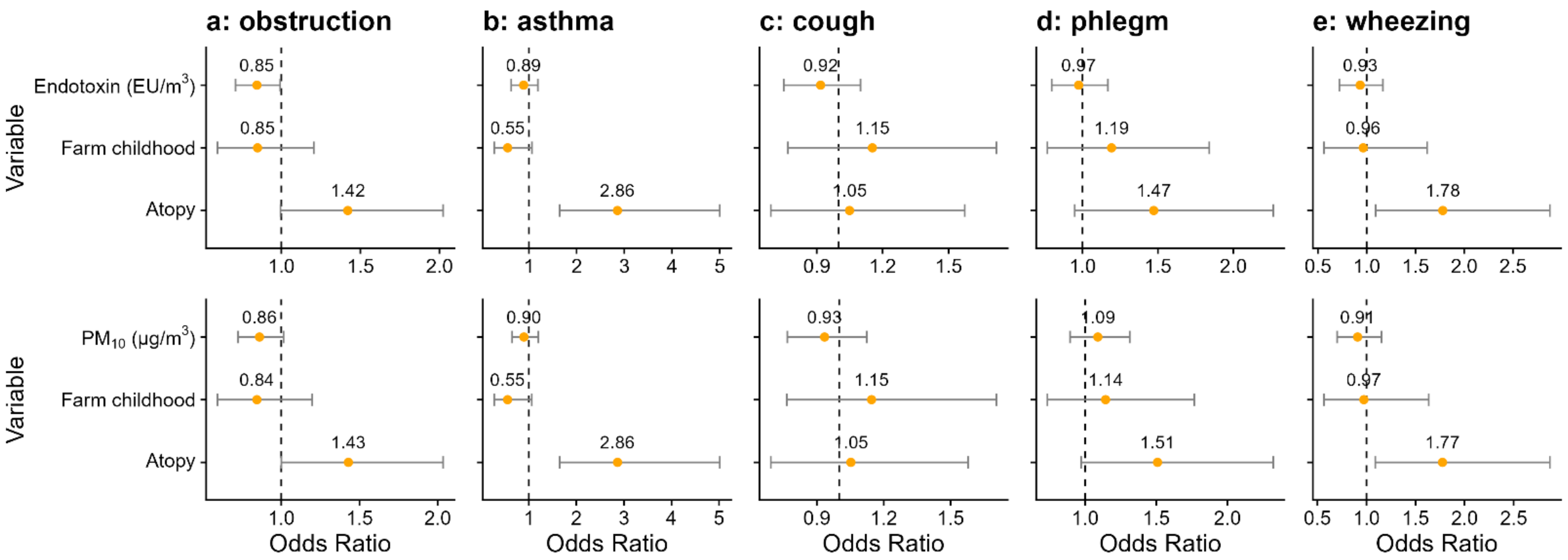

Supplementary figure 2: Logistic regression model associations between livestock-related air pollutants and a: new onset airway obstruction, defined as T_0 FEV1/FVC > 0.7 and T1 FEV1/FVC < 0.7; b: self-reported doctor diagnosed asthma; c: chronic daily cough > 3 months last year; d: chronic daily phlegm >3months last year; e: wheezing last year.

Note: all variables have been mutually adjusted. Models were additionally adjusted for age, sex, height, BMI, education level and smoking history. Predicted livestock-related air pollutant concentrations at residential addresses were winsorized to the 99.5 percentile.
